# Refractory Acute Interstitial Nephritis in the Setting of Nivolumab Therapy

**DOI:** 10.1155/2021/6640154

**Published:** 2021-03-01

**Authors:** Antonio Faieta, Tavis Dancik

**Affiliations:** ^1^Department of Internal Medicine, Beaumont Health System, Royal Oak, MI, USA; ^2^Department of Nephrology, Beaumont Health System, Royal Oak, MI, USA

## Abstract

A 65-year-old male patient with metastatic CCRCC developed steroid-dependent, grade 3 AIN secondary to nivolumab weeks after its initiation that resulted in 3 hospitalizations with acute renal failure. The patient was started on MM and his AIN was successfully controlled after a 2-year period of follow-up. Refractory renal AIN resulting from PD-1 inhibitor use is rare, and its successful treatment with mofetil mycophenolate with a 2-year follow-up in a patient with metastatic CCRCC has not been reported. This case is important because not only was his renal irAEs controlled but also long-term treatment with MM did not result in progression of metastatic disease.

## 1. Introduction

A 65-year-old male with a past medical history of essential hypertension, type 2 diabetes mellitus, obesity with a BMI of 31.65 kg/m^2^, stage 2 chronic kidney disease (CKD), COPD, paroxysmal atrial fibrillation, dyslipidemia, and history of urinary retention secondary to benign prostate hyperplasia was found to have a 9.5 × 6 × 7.1 cm enhancing mass in the left kidney after an abdominal CT scan with intravenous contrast done for an R renal cyst follow-up. A high-resolution CT scan of the chest (HRCT) revealed numerous nodules suspicious for metastases. A CT scan-directed lung biopsy revealed metastatic clear cell renal cell carcinoma (CCRCC). One month later, the patient underwent a left nephrectomy and lymphectomy followed by sequential therapy with sunitinib, everolimus, and bevacizumab, which overall controlled the disease for a total period of 31 months. However, his disease eventually progressed, and new brain metastasis was found. The patient underwent gamma knife radiosurgery and was started on nivolumab, a checkpoint inhibitor (CPI). Two months later, around his fourth cycle of nivolumab, the patient's creatinine was found to be 1.6 mg/dl, from his baseline of 1.0–1.2 mg/dl. Four months after nivolumab was started, following his 8th cycle of nivolumab, the patient was admitted to the hospital because a creatinine level of 4 mg/dl was noted. The patient had recently completed a course of cephalexin for a throat infection and was on omeprazole, but not NSAIDs or ACEi. On admission, creatinine was 6.09 mg/dl and BUN was 68 mg/dl, and there were metabolic acidosis and hyperkalemia. There was evidence of urinary retention on kidney and bladder ultrasound based on a PVR of 450 cc. Urinalysis revealed few eosinophils, 3–10 leukocytes, and subnephrotic range proteinuria. Complement levels were normal. The patient was started on intravenous hydration, omeprazole was discontinued, and a Foley catheter was placed. However, his renal function did not improve. The patient was subsequently started on 45 mg of prednisone orally twice daily for presumptive acute interstitial nephritis (AIN) secondary to nivolumab. He was discharged on a prednisone taper and his renal function continued to improve.

Two months later, close after ending his prednisone taper, the patient presented to the ER complaining of 2 weeks of worsening shortness of breath and 20-pound weight gain. On admission, physical examination revealed jugular venous distension and 4+ lower extremity pitting edema up to the knee, but no lung rales. Creatinine was 7.79 mg/dL, BUN 81 mg/dL, and potassium 6.4 mg/dL. Urinalysis revealed eosinophiluria, subnephrotic range proteinuria, and leukocyturia >50. Urinary retention was ruled out. Urine cultured grew 100,000 CFU of *E. coli*. Chest X-rays revealed signs of lung congestion and new small bilateral pleural effusions. A transthoracic echo demonstrated severely enlarged left atrium, mild to moderate tricuspid regurgitation, evidence of mildly elevated right ventricular systolic pressure, and trace pulmonary regurgitation that were not present on a TTE done 2 years prior. Left ventricular chamber size was normal, there were no distinct wall motion abnormalities, and a visually estimated ejection fraction was 55%. He was started on furosemide and antibiotics, but his renal function continued to worsen. The presumptive diagnosis of recurrent AIN secondary to nivolumab was made, and the patient was started on a pulse IV methylprednisolone followed by oral prednisone. After creatinine peaked to 8.29 mg/dL, the patient was discharged one week later with a creatinine level of 3.74 mg/dL on a prednisone taper and his creatinine continued to improve weeks later.

**Figure 1:**
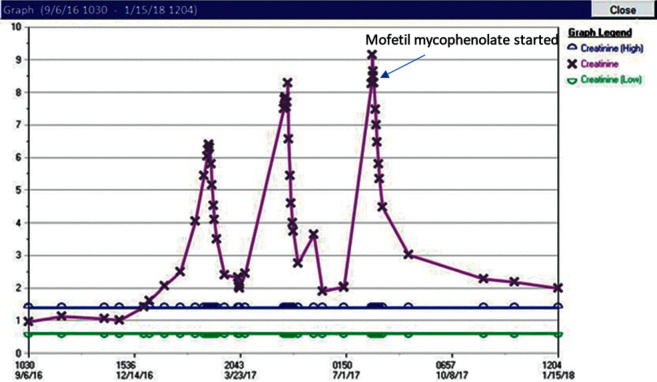
Patient's acute kidney injury course. The first 2 creatinine peaks represent acute kidney injury during steroid tapering. Note that when mofetil mycophenolate was initiated, the patient's renal function improved and eventually stabilized, and complete steroid weaning was possible.

Few weeks after ending his second steroid taper, the patient was hospitalized for the third time because of a creatinine level of 8.27 mg/dL. There were eosinophiluria, proteinuria, significant leukocyturia, metabolic acidosis, and hyperkalemia with peak T waves on EKG. The patient was deemed to be steroid-dependent, and consideration to a steroid-sparing drug was given. The patient was started once again on pulse 500 mg IV methylprednisolone and 1 gram of mofetil mycophenolate (MM) twice daily. Renal biopsy was finally done and showed moderate interstitial inflammation mainly composed of lymphocytes along with secondary acute tubular injury. In addition, trichrome staining revealed a moderate amount of interstitial fibrosis and tubular atrophy. No evidence of glomerulonephritis or crescent formation was present. On immunofluorescence, glomeruli were negative for IgG, IgA, IgM, C3, kappa, lambda, and C1q. Renal function improved, and the patient was discharged on a prednisone taper and mofetil mycophenolate. In the following months, the patient was able to complete his prednisone taper and his creatinine remained stable on his new baseline, 2 mg/dL, and proteinuria, leukocyturia, and proteinuria resolved. The entire course of his acute kidney injury and response to treatment is depicted in Figure 1. His dose of mofetil was eventually weaned to 250 mg twice daily which he is presently on. Overall, the patient only received 8 cycles of nivolumab. PET CT after 3 years off nivolumab and while receiving MM revealed no new or progressive lesion, decreased avidity in the only avid lesion in a subcarinal lymph node, and stable brain lesion. The patient is currently doing well.

## 2. Discussion

Nivolumab, a checkpoint inhibitor, is a human IgG4 anti-PD-1 antibody that selectively blocks the interaction between PD-1 expressed in activated Th lymphocytes and its ligands, PD-L1 and PD-L2, expressed in tissues and cancer cells [1]. PD-L1 and PD-L2 expressions in tissues are one of the major determinants of peripheral immune tolerance. PD-L1 is overexpressed in tissues via lymphokines during states of T-cell activation as a means to safeguard themselves from being “attacked.” Furthermore, some tumors constitutively express PD-L1 which dampens the activation of tumor-infiltrating lymphocytes. By blocking the interaction between PD-1 and PD-L1, the T-cell response is unleashed against the tumor. CCRCC expresses PD-L1 and there lies the rationale for nivolumab use. Nivolumab was approved by the FDA for its use in metastatic CCRCC in 2015 after demonstrating a significantly superior objective response rate and overall survival compared to everolimus in patients previously treated with bevacizumab [2].

Sometimes, T cells turn against a person's own tissues due to loss of peripheral tolerance induced by CPIs, resulting in immune-related adverse events (irAEs). Overall, irAEs are common in patients on CPIs and can affect almost any organ. CPIs are indicated for the treatment of several metastatic cancers. As cancer is more common with increasing age, the typical patient receiving CPIs is in the elderly age group and has numerous comorbidities and thus limited organ reserve, as the patient in this case vignette. IrAEs' timing of presentation and severity are vastly heterogeneous but more frequently occur between 21 and 245 days after they are initiated [3]. Commonly, irAEs present grade 1-2 events (at most requiring only symptomatic management), but grade 3 or 4 events (organ- or life-threatening) can occur in up to 15% of the patients that merits discontinuation of CPIs [4]. However, there are not practical ways for clinicians to predict the degree of an irAE's severity and refractoriness early on its course, when the affected organ is at its best chance of recovery. In addition, most of the irAEs are reversible with treatment.

Renal irAEs can occur with CPIs. The patient can present with variable elevation in creatinine, leukocyturia, and subnephrotic range proteinuria [5, 6]. Case reports and series of renal irAEs, including this case vignette, patients commonly had numerous risk factors for and/or an already established CKD. Furthermore, they were frequently receiving medications typically associated with drug-related AIN, such as proton pump inhibitors or first-generation cephalosporins. CKIN recommends monitoring creatinine frequently early in the initiation of CPIs as irAEs tend to occur more frequently during this period [7]. AIN is the most common renal irAE [6]. However, pathologic specimens have shown that renal irAEs are histologically heterogeneous and may inform about renal outcomes and treatment response. A higher degree of interstitial lymphocytic infiltrate and acute tubular damage may predict loss of renal function while a higher degree of granulomatous necrotizing vasculitis and glomerulonephritis (GN) seems to portend better renal outcomes [3, 6]. Furthermore, in a case series of 16 patients with renal irAEs, who were treated initially with a course of steroids and CPI discontinuation, none of the 5 AIN cases had complete renal function recovery and 2 of them ended up in permanent hemodialysis. Those with GN had a variable response to treatment depending on the type of GN. Those with granulomatous GN had complete recovery while IgA GN did not respond to steroids or MM [6]. Because of the severe renal irAEs' rareness, there are no guidelines for their management. Most of the information about the management of renal irAEs comes from case reports and series in which glucocorticoids were favored as front-line therapy [3, 6, 8, 9]. Overall, it seems that patients who were older, who developed AIN close to the start of CPIs, who had a high creatinine elevation peak, had metastatic disease, and those undergoing concurrent treatment with nivolumab and ipilimumab, present with more aggressive AIN phenotype characterized by severe lymphocytic AIN and tubular injury that may benefit from early and prolonged steroid-sparing immunosuppression. In addition, long-term use of immunosuppressive medication to treat CPI high-grade adverse effects has not been associated with worse prognosis or overall survival [5]. On the other hand, the prognosis of a patient with CKD stage 2 that progresses to stage 4 (our patient) after an irAE may potentially be worse. Thus, early biopsy and immunosuppression may decrease the risk of irreversible kidney damage, particularly in patients with grade 3-4 renal irAEs and those with low physiological reserve such as the elderly or patients with comorbidities. It is important to notice that, in this case, biopsy was delayed under the presumption of nivolumab-induced interstitial nephritis which missed the severity of the histologic phenotype and other potential differential diagnoses. The duration of immunosuppression is unknown, but our patient did well 3 years on MM and off nivolumab.

Also, it is unknown whether immunosuppressive medication can be used concomitantly with CPIs to control irAEs. Interestingly, a report of an elderly patient with metastatic anal melanoma was treated with a sequential use of ipilimumab followed by nivolumab after which she developed severe steroid-dependent AIN. This patient was kept on nivolumab concurrently with maintenance prednisone at a dose of 10 mg, and her renal function improved and stabilized. When prednisone was stopped, she developed a recurrence of AIN and nivolumab had to be discontinued. Of note, most of her metastatic lesions demonstrated ongoing regression except for new brain lesions treated with stereotaxic radiation after nivolumab was discontinued [9]. Some patients will only need a course of steroids and discontinuation of CPIs to treat their AIN [10]. In a series of 13 cases of AKI secondary to CPIs, AIN was the most common histological finding; the most common cancer treated was metastatic melanoma; AIN was more common in patients treated with nivolumab alone, ipilimumab alone, and twice as common with nivolumab and ipilimumab combination compared to pembrolizumab; half of the patients partially recovered their renal function with steroids, less than a quarter had a complete response, and one patient showed TMA and did not respond to steroids [3]. In this case series, those patients treated conservatively did not have an improvement in renal function. One patient required mofetil mycophenolate because of worsening renal function on methylprednisolone.

CPIs have proven to be effective in the management of some advanced cancer, so their use is expected to increase as well as the incidence of irAEs and physicians will have a major role in diagnosing and treating them with the goal of preventing irreversible organ damage that can increase morbidity and affect prognosis. This case is relevant because it presents a patient with a refractory grade 3 renal irAEs that was effectively controlled with long-term use of mofetil mycophenolate with ongoing regression of his metastases. Finally, to change the current steroid-centered paradigm, there is a need for randomized trials that compare initial treatment with long-term treatment with steroid-sparing immunosuppressants with tapering courses of steroids in a patient with grade 3-4 irAEs and high comorbidity burden.

## Data Availability

No data were used to support the findings of this study.
